# Current Treatment Strategies for Diffuse Tenosynovial Giant Cell Tumor: A Review of the Literature

**DOI:** 10.2106/JBJS.OA.25.00313

**Published:** 2026-01-13

**Authors:** Hannah Mosher, Kristen Dean, Gabrielle Meli, Jessyka Desrosiers, Brooke Crawford, H. Thomas Temple, Francis J Hornicek, Andrew E. Rosenberg, Emily Jonczak, Emanuela Palmerini, Erik J. Geiger

**Affiliations:** 1 Department of Orthopaedic Surgery, University of Miami Miller School of Medicine, Miami, Florida; 2 Sylvester Comprehensive Cancer Center, University of Miami Health System, Miami, Florida; 3 Department of Pathology, Miller School of Medicine, Miami, Florida; 4 Department of Medicine, Division of Medical Oncology, Miller School of Medicine, Miami, Florida

## Abstract

**Background:**

Diffuse tenosynovial giant cell tumor (DTGCT) is a locally aggressive benign tumor of the synovium. Patients often initially present with pain, stiffness, and swelling of the affected joint with varying levels of severity. Treatment traditionally involved surgical resection exclusively; however, this could be complicated by high disease recurrence rates. New research has introduced several targeted systemic therapies onto the market changing the treatment paradigm and necessitating a multidisciplinary treatment approach in specialized centers to optimize patient outcomes.

**Methods:**

This review synthesizes the current literature on DTGCT including its pathophysiology, classification, diagnosis, and available treatment options. There is a particular focus on the newer systemic therapies available and how these medications may be used in conjunction with surgery to enhance disease control.

**Results:**

DTGCT most commonly affects young to middle-aged adults, with a slight female predominance, and is most frequently found in the knee. Arthroscopic and even open synovectomy can have disease recurrence rates exceeding 50%. Colony stimulating factor 1 (CSF1) receptor inhibitors have proven effective at symptom palliation and reducing tumor burden in approximately 40% of patients. While these medications improve the quality of life for patients with unresectable disease, they may also be effective in the neoadjuvant setting to downstage surgical approaches and possibly improve disease control in otherwise highly morbid cases.

**Conclusions:**

Surgery alone, the traditional standard for DTGCT, is often insufficient due to high recurrence rates. Systemic therapies can restore function and improve quality of life in patients with advanced disease with rare—but potentially serious—adverse effects. Combining surgical resection with neoadjuvant CSF1R inhibition may provide superior outcomes. Further research is needed to refine the role of systemic agents and develop multidisciplinary protocols. Although initial symptoms often lead patients to community providers, optimal care for patients with DTGCT is best delivered at referral centers with dedicated musculoskeletal oncology programs.

**Level of Evidence:**

Level V. See Instructions for Authors for a complete description of levels of evidence.

## Introduction and Background

Diffuse tenosynovial giant cell tumor (DTGCT) is a rare, benign, yet locally aggressive neoplasm arising from the synovial lining of joints, tendons, and bursae. Formerly known as pigmented villonodular synovitis, DTGCT is characterized by diffuse involvement of the synovium with ill-defined margins and infiltrative growth patterns^1^. Clinically, it often presents with insidious onset of joint pain, swelling, stiffness, and limited range of motion, which can progress to joint arthrosis and significant functional impairment^2^. The knee is the most affected joint, followed by the hip, likely due to the extensive synovial surfaces within these joints provide^2-4^.

Historically, surgical resection was the mainstay of treatment for DTGCT^4^. This approach was largely adopted due to the tumor's potentially aggressive behavior and its local effects on joint function, with the goal of removing the abnormal synovial tissue to alleviate symptoms and prevent further joint destruction^5,6^. However, despite complete or near-complete synovectomy, recurrence rates have remained high, reportedly over 50% in some series, often necessitating repeat surgeries^2,4^. These repeat interventions increase the risk of surgical morbidity—including joint instability or stiffness—and recurrent tumor increases the need of therapeutic total joint arthroplasty.

The identification of the colony stimulating factor-1 (CSF1) gene rearrangement as a molecular driver of DTGCT pathogenesis has shifted the treatment paradigm^7,8^. This discovery led to the development of targeted systemic therapies aimed at interrupting the CSF1/CSF1R (Receptor) signaling axis responsible for tumor proliferation and the associated inflammatory microenvironment. As a result, systemic therapies have emerged as a critical adjunct or alternative to surgery, particularly in patients for whom surgery is considered highly morbid. This review summarizes the current state of the field and particularly focuses on the roles systemic therapy and surgical resection play in disease control, aiming to optimize patient outcomes.

## Epidemiology

DTGCT primarily affects young adults, peaking in the fourth decade, with an incidence of 4 to 8.4 cases per million person-years and a slight female predominance (51%-55% of cases)^5^. Approximately 80% of cases occur in the knee joint followed by the hip (15%), ankle, and elbow^5,6,9^. Local recurrence rates after surgical resection range from 20 to 50% with higher recurrence observed in diffuse-type lesions.^10,11^.

## Pathophysiology

DTGCT is a benign synovial neoplasm driven by a chromosomal translocation t (1:2) (p13; q37), resulting in fusion of the COL6A3 promoter region with the *CSF1* gene, leading to CSF1 overexpression^12,13^. This promotes CSF1 dependent recruitment of macrophages, lymphocytes, and multinucleated giant cells which ultimately constitute up to 90% of the tumor mass^13,14^. Recent studies have identified secondary mutations in p53, phosphatase and tensin homolog, and Kirsten rat sarcoma viral oncogene homolog in recurrent tumors, highlighting the potential role of genomic instability in advanced disease^14^.

Cellular crosstalk plays another significant role in the pathophysiology of DTGCT where neoplastic synovial cells and macrophages engage in paracrine signaling. Activation of the CSF1R stimulates macrophage survival and polarizes these cells toward an M2-like, protumorigenic phenotype^7,8^.

## Classification

Tenosynovial giant cell tumors are classified into diffuse or localized types which guide prognosis and therapy. Localized tumors are characterized by well-demarcated nodules with minimal synovial involvement and are associated with low recurrence after surgical resection^6^. Diffuse type tumors are characterized by more aggressive, infiltrative growth, larger size, synovial hyperplasia, and high recurrence rates following surgical excision^15^.

## Imaging

Magnetic resonance imaging (MRI) is the primary modality for evaluating extent of disease.^16^. Pathognomonic findings of DTGCT include low signal intensity on T1-weighted and T2-weighted images and the appearance of a blooming artifact on gradient-echo sequences due to hemosiderin deposition^17,18^. MRI can also be used to differentiate between localized and diffuse growth patterns with cartilage involvement demonstrating a specificity of 100% in intra-articular and 88.6% in extra-articular cases^18^. Radiographs may provide additional insight and are valuable for evaluating concomitant joint arthritis and cartilage loss in advanced cases.

## Diagnosis

DTGCT is best diagnosed via core-needle biopsy, which may be ultrasound or CT-guided^19-21^. While clinical presentation and imaging may provide diagnostic insights, biopsy remains essential for histologic confirmation and definitive diagnosis, ultimately guiding management. Histologically, both localized and diffuse types show neoplastic mononuclear cells that have abundant often eccentric eosinophilic cytoplasm, osteoclast-like multinucleated giant cells, foamy macrophages, hemosiderin deposition, and varying amounts of stromal sclerosis (Figs. 1-A and 1-B). Diffuse lesions are further characterized by infiltrative margins and a villonodular architecture. Co-existing hemosiderosis synovitis is commonplace^22^. Furthermore, immunohistochemistry can assist in distinguishing DTGCT from other synovial pathologies with key markers, including Clusterin, Podoplanin, receptor activator of nuclear factor kappa-B ligand, and glutamine fructose-6-phosphate transaminase 2^23-26^.

**Fig. 1-A:**
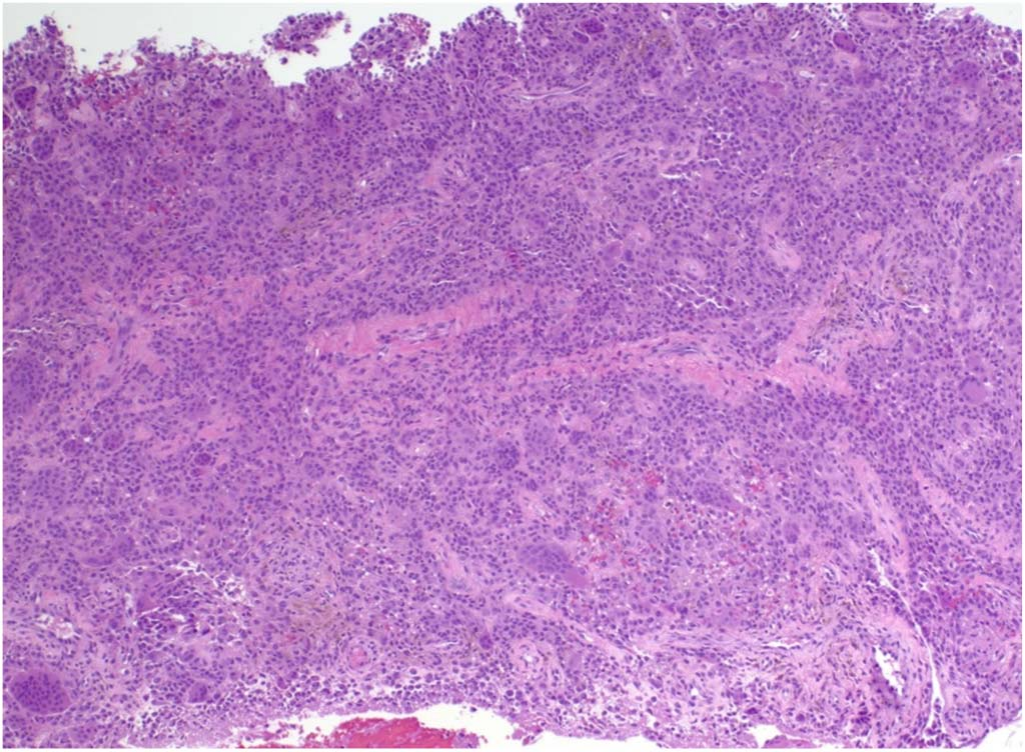
DTGCT is hypercellular and composed of different cell types.

**Fig. 1-B:**
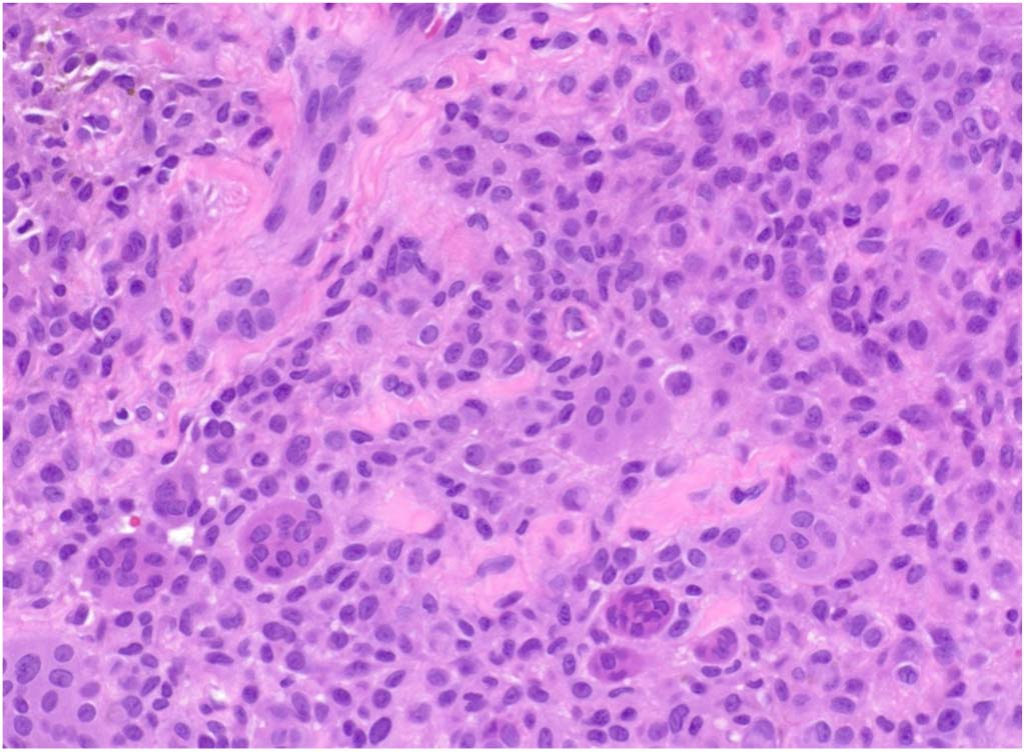
Neoplastic mononuclear cells are polyhedral with abundant eosinophilic cytoplasm and are admixed with nonfoamy macrophages and scattered osteoclast-like giant cells. DTGCT = diffuse tenosynovial giant cell tumor.

## Treatment

### Surgical Intervention

The standard first-line treatment for symptomatic DTGCT is surgical excision with the goal of complete tumor removal. Surgery may be performed open or arthroscopically; however, arthroscopic-only excision is associated with high local recurrence, estimated as high as 56%^25^. In a study of 966 surgically treated patients, 44% had recurrent disease at a median follow-up of 54 months, and recurrence-free survival rates were 62% at 3 years, 55% at 5 years, and 40% at 10 years^9^. An international, multicenter, retrospective cohort study by Mastboom et al. found a 5-year recurrence-free survival rate of 66% for open surgery vs. 54% for arthroscopic surgery, although selection bias complicates this comparison^9^. Furthermore, a recent meta-analysis by Chandra et al., found a 1.56-fold increased risk of recurrence in DTGCT of the knee when treated via an arthroscopic approach^27^. A randomized control trial assessing recurrence in arthroscopy vs. open surgery has not been conducted.

Although arthroscopy allows for faster recovery times, visibility can be limited during resection compared with open surgery, and subtotal excision increases the recurrence risk^28^. Overall, the selection of the surgical technique is individualized based on the patient's clinical presentation, ideally incorporating input from multiple subspecialties, with the primary goal of achieving complete tumor removal. Irrespective to surgical method, incomplete resection is an independent risk factor for recurrence^29^.

More recently, combined approaches using both open and arthroscopic techniques have been used to maximize local control while minimizing invasiveness and associated morbidity. Combined approaches typically include an arthroscopic synovectomy anteriorly with an open approach posteriorly to remove intracapsular and extracapsular disease with studies showing low rates of recurrence and favorable functional outcomes^30,31^. When combined approaches are used, it is essential to have experts in arthroscopy involved, typically orthopaedic sports medicine surgeons, alongside orthopaedic oncology surgeons, to ensure adequate resection in both the intra-articular and extra-articular spaces while minimizing surgical morbidity and maximizing joint preservation^32^.

### Radiation Therapy

Radiation therapy (RT) alone has historically played a role in minimizing or delaying the locoregional recurrence of disease. The role of RT has been established in the postoperative setting, particularly after incomplete resection, to provide local disease control or limb salvage in cases of recurrent disease^33,34^. Despite such potential benefits, there is limited clinical use of RT due to the long-term adverse outcomes associated with this treatment exposure, including the risk of functional impairment, avascular necrosis, and/or malignant transformation^35^. With increasing use of systemic therapies, RT is now reserved for select refractory cases^36^.

### Systemic Therapies

#### Imatinib and Nilotinib

Given the high rate of disease relapse, compromised physical mobility, and reduced quality of life, alternatives to surgery have been sought. Imatinib and nilotinib are 2 small molecule multityrosine kinase inhibitors with limited CSF1R inhibition that have been trialed off-label for treatment of DTGCT, with limited efficacy^37^. They are a multicenter phase II clinical trial enrolled 56 patients across 11 cancer centers to evaluate the off-label use of nilotinib in treating DTGCT not amenable to surgery^38^. Ninety-six percent of patients experienced treatment-related adverse events, raising concerns about tolerability^39^. At final follow-up, 52.1% of patients experienced disease progression, with a median progression-free survival (PFS) of 77 months. Notably, patients who completed the full 12-month treatment protocol demonstrated a 5-year PFS rate of 71.5%. While nilotinib demonstrated short-term disease control, the high incidence of adverse effects and long-term risk of recurrence underscore its limitations.

Similarly, a multi-institutional retrospective study published in 2012 evaluated the off-label use of imatinib in 29 patients, reporting a moderate therapeutic benefit. Among the cohort, one patient achieved complete remission, 4 exhibited partial responses, and 18 patients maintained stable disease. The remaining 6 patients of the starting sample discontinued imatinib due to clinically significant toxicity^40,41^. A separate study involving 25 patients with locally advanced or recurrent DTGCT observed a moderate reduction in tumor volume, confirmed by MRI and positron emission tomography-computed tomography imaging^42^. Despite this reduction in tumor size, 80% of patients reported adverse effects, including fluid retention, skin rashes, fatigue, and nausea. In addition, 3 patients experienced serious complications such as elevated creatinine levels, neutropenic sepsis, and liver dysfunction^42^. The National Comprehensive Cancer Network (NCCN) guidelines as of 2022, classified imatinib and nilotinib as category 2a drugs, indicating it may be useful in certain clinical scenarios, though it is not broadly recommended for all patients^43^. These limitations shifted focus to designing medications with more potent and targeted inhibition of CSFR1.

#### Pexidartinib

Pexidartinib, Food and Drug Administration (FDA)-approved in 2019, was the first drug specifically approved for the treatment of adults with DTGCT and became the mainstay of systemic therapy^44^. In contrast to imatinib and nilotinib, pexidartinib has a category one preferred recommendation by the NCCN guidelines, and it has been deemed an appropriate intervention for all TGCT cases based on high-quality evidence. The real-world efficacy of pexidartinib treatment is demonstrated through histological images (Figs. 2-A and 2-B) from a patient with DTGCT treated at our institution.

**Fig. 2-A:**
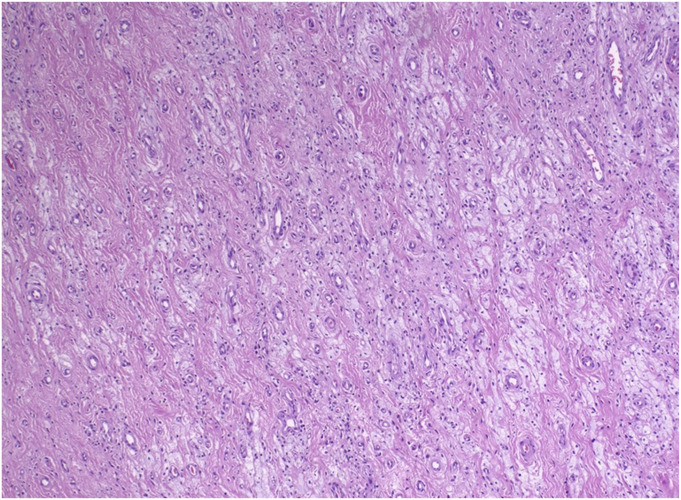
Area of DTGCT treated with pexidartinib showing diminished cellularity and more prominent collagenous stroma. This patient received 250 mg of pexidartinib BID by mouth and reported improvement in their presenting symptoms within days and no reported side effects during their treatment course (9+ months) thus far.

**Fig. 2-B:**
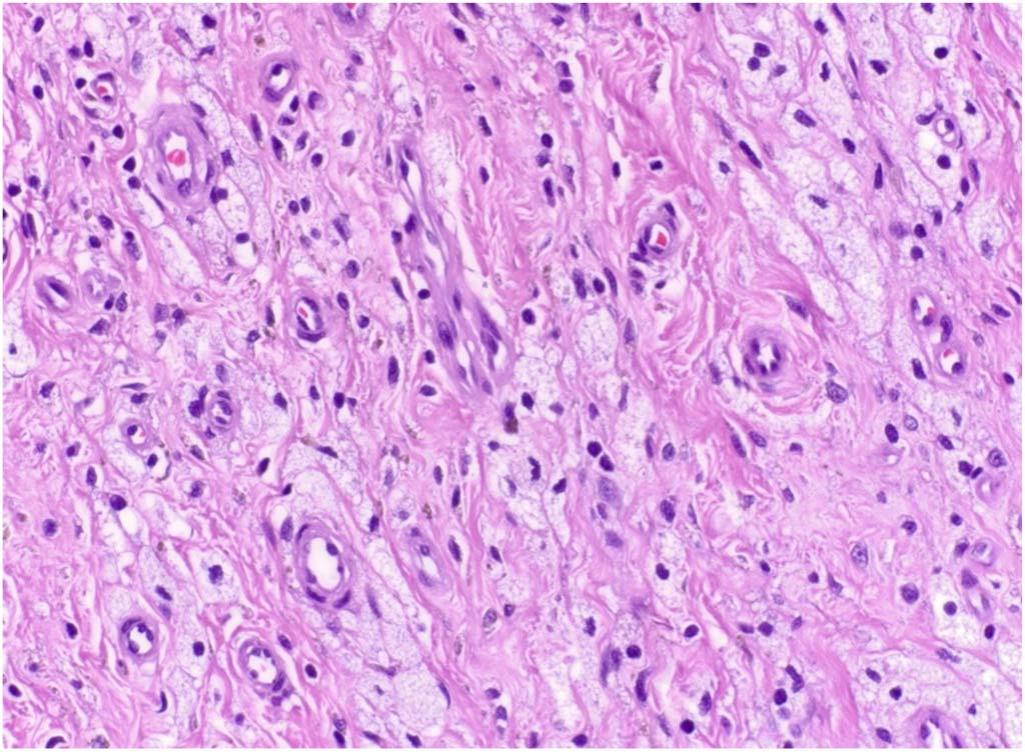
There has been dropout of tumor cells and macrophages leaving behind groups of foamy histiocytes enmeshed in a collagenous stroma. DTGCT = diffuse tenosynovial giant cell tumor.

The phase III placebo-controlled randomized clinical trial ENLIVEN evaluated the risks and benefits of pexidartinib^40^. Conducted across 13 countries, the trial enrolled patients who were randomized in a 1:1 ratio to receive either pexidartinib or placebo over a 24-week double-blind period. The primary endpoint was the overall response rate (ORR) as assessed by Response Evaluation Criteria in Solid Tumors v1.1 criteria, while secondary endpoints included range of motion, patient-reported outcomes, stiffness, pain, and tumor volume score. Pexidartinib demonstrated a statistically significant difference in ORR compared with placebo (39% vs. 0%, p < 0.0001), along with clinically meaningful improvements in physical function and symptom relief. Specifically, patients who received the drug reported significantly greater improvements in joint range of motion, reductions in stiffness and pain, and enhanced quality of life as measured by PROs. In addition, the study found a significant reduction in tumor volume, supporting the correlation between tumor burden and symptom severity in DTGCT. The ENLIVEN study also highlighted substantial safety concerns with nearly all patients (98%) in the pexidartinib group experiencing at least one treatment-emergent adverse event (TEAE) and 51% experiencing adverse effects that led to dose modification or treatment interruption. The most common TEAEs included hepatotoxicity, nausea, pruritis, fatigue, and hypertension^40^. These findings underscore the importance of careful patient selection and close monitoring when considering pexidartinib for DTGCT management.

A cross-sectional study of 83 respondents taking pexidartinib (yielding a 69.2% response rate) for DTGCT sought to elicit the patients' perspectives on treatment^45^. Compared with symptoms before treatment, the majority of patients (78.3%) reported improvement in overall DTGCT symptoms, including decreased pain and stiffness. In addition, there was a significant decrease in the use of opioids, NSAIDs, corticosteroids, and physical therapy during treatment, which aligned with patient-reported symptom improvement^45^. Although limitations of cross-sectional studies include response bias due to self-reported data, these findings are congruent with the results of the ENLIVEN study^46^. These findings offer valuable insight into the patient experience in clinical practice, providing evidence for leveraging the benefit of pexidartinib in improving quality of life and functional capabilities^45^.

#### Vimseltinib

Vimseltinib—an oral CSF1R kinase inhibitor—was approved by the FDA in February 2025 for use in cases of patients with symptomatic DTGCT where surgical resection would be considered severely morbid^47,48^. Vimseltinib (CDD-3014) binds to CSF1 receptors with a specificity of more than 100-fold compared with other similar kinases and had been proven to decrease infiltrating macrophages and CD16^+^ monocytes in preclinical studies^49^.

The MOTION trial was a double-blind, multicenter, randomized (2:1), and placebo-controlled study evaluating the efficacy of vimseltinib for patients with symptomatic DTGCT. ORR to the systemic therapy was assessed through blinded, independent radiological review at the 25-week mark of the study^50^. Results revealed effective reduction in tumor volume in approximately two-thirds of the treatment group, compared with no reduction of tumor volume in the placebo group. Associated reduction in DTGCT symptoms were also reported, including improved physical function, range of motion, joint stiffness, pain, and overall health^50^. In addition, in comparison with pexidartinib, vimseltinib presented fewer severe side effects and no evidence of liver injury, suggesting a potential opportunity for this drug to serve as maintenance therapy for patients concerned with recurrence of DTGCT^49^.

Despite its current indication primarily for nonoperative patients, there is strong potential for expanding the approved uses of vimseltinib as Phase IV clinical trials are conducted. Future insights from broader clinical application and postmarketing studies may further clarify vimseltinib's role, including the potential for combination use alongside surgical intervention^50^.

## CSF1R Inhibitors and Surgery

DTGCT presents significant challenges due to high recurrence rates, and repeat resections are associated with increased surgical morbidity. While systemic therapies may provide moderate local disease control, they are unable to restore joint function already lost^51^. As such, combining systemic therapies with surgical intervention may offer improved disease clearance rates and quality of life outcomes^51,52^. Geiger et al. published an initial case report describing such dual therapy for DTGCT, using pexidartinib in the neoadjuvant setting to decrease DTGCT disease burden conjunction followed by resection and upper extremity limb reconstruction^51^. Although pexidartinib was initially FDA-approved for patients who are not surgical candidates, this case explored its potential synergistic benefit alongside surgery for a patient with widely recurrent disease. In this case, neoadjuvant pexidartinib facilitated subtotal but near-complete disease resection and limb salvage surgery. The patient was maintained on pexidartinib postoperatively given the small amount of remaining disease in the axilla^51^.

Building on this, Bernthal et al. reported an additional 3 DTGCT cases treated with a combination of surgery and systemic therapy. Each case involved a different anatomical site—hip, foot, knee with the hip only receiving neoadjuvant pexidartinib treatment while the knee and foot cases received both preoperative and postoperative pexidartinib therapy. By the final 2 years after treatment initiation, the disease was deemed fully eradicated. In all cases, serial MRI scans (beginning at 3 months after baseline and repeated at 6-month intervals) were used to monitor therapeutic response and disease status^52^.

**Fig. 3:**
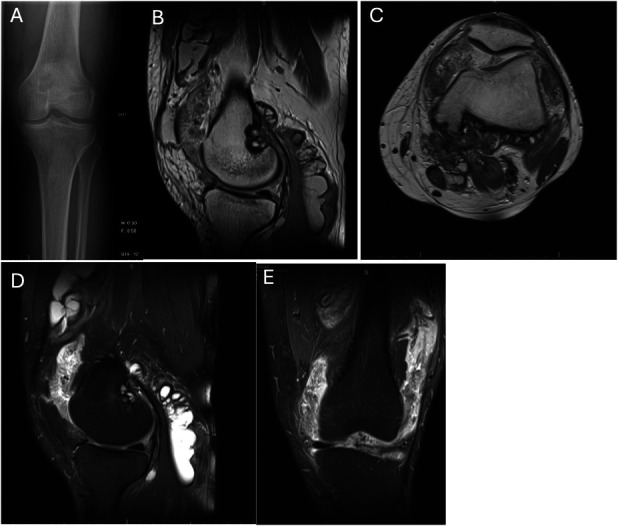
Pretreatment imaging of patient treated with both pexidartinib and surgery. **Fig. 3-A** Anterior posterior radiograph of the knee demonstrates mild medial joint space narrowing without osseous arthritic changes. Normal alignment. There is focal osteolysis evidence in the superior aspect of the medial femoral condyle. **Fig. 3-B** T1-weighted sagittal and axial (**Fig. 3-C**) MRI of the knee demonstrating massive intra- and extra-articular tumor burden extending from the suprapatellar pouch anteriorly to the gastrocnemius tendon bursa posteriorly. There is progression into the posterior aspect of the distal femur. **Fig. 3-D** T2-sagittal MRI highlighting the massive joint effusion and postcontrast enhancement (**Fig. 3-E**) of the diffuse synovial tumor throughout every knee compartment extending to the popliteal fossa. MRI = magnetic resonance imaging.

**Fig. 4:**
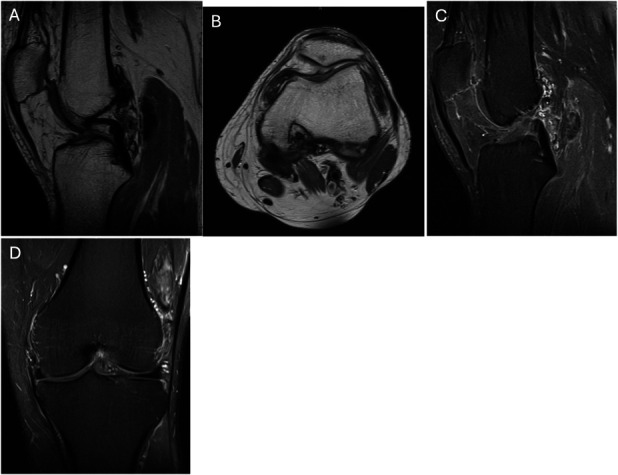
Imaging after neoadjuvant pexidartinib treatment. **Fig. 4-A** T1-weighted sagittal and axial (**Fig. 4-B**) with postcontrast, fat-saturated sagittal (**Fig. 4-C**) and coronal (**Fig. 4-D**) demonstrating substantial but incomplete disease response. There are persistent foci of TGCT anteriorly in the medial and lateral gutters, as well as posteriorly along the joint capsule and into the gastrocnemius muscle belly. The lytic defect of the posterior distal femur is clearly demonstrated. TGCT = tenosynovial giant cell tumor.

**Fig. 5:**
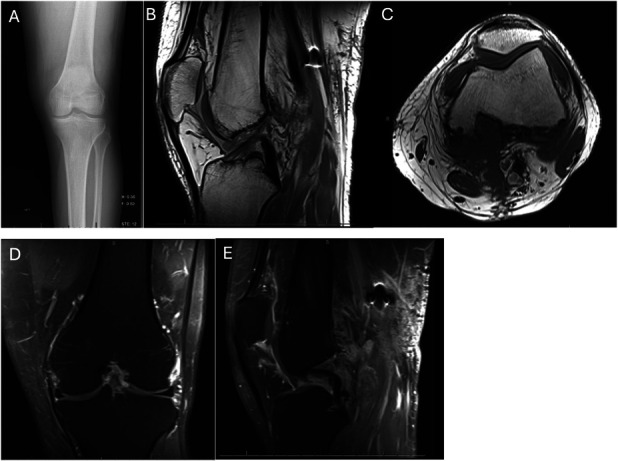
Postoperative imaging for patient after anterior arthroscopic and open posterior excision of tumor. **Fig. 5-A** Anterior posterior radiograph of the knee with only notable changes from preoperative imaging being curetting and bone grafting of the medial condylar defect. **Fig. 5-B** T1-sagittal and axial (**Fig. 5-C**) MRI demonstrating near complete disease resection with bone grafting of the posterior condylar defect. **Fig. 5-D** Coronal T2 and sagittal postcontrast (**Fig. 5-E**) MRI highlighting removal of the anterior and posterior intra- and extra-articular disease burden. MRI = magnetic resonance imaging.

Early studies suggest that neoadjuvant CSF1R inhibitors hold promise in managing DTGCT, even in cases initially deemed resectable. Preoperative use may downstage disease, allowing less invasive surgery with comparable disease control, and may render some inoperable cases operable. A case from our institution similarly demonstrates meaningful tumor reduction with preoperative and postoperative pexidartinib therapy (Figures 3–5). The patient received pexidartinib at the recommended dosing of 250 mg twice daily (BID) for ∼7 months before surgery. The patient was asked to discontinue pexidartinib ∼2 weeks before surgery due to preoperative protocols, but then following surgery, Pexidartinib was resumed at the same dose of 250 mg BID. Monthly lab draws were completed to monitor for liver toxicity, a known side effect of pexidartinib with no abnormalities found thus far. As highlighted by this case and others, as newer agents with improved safety and tolerability emerge, their perioperative use may further reduce surgical morbidity and the need for repeat interventions.

## Conclusion

Symptomatic DTGCT has previously been treated exclusively with arthroscopic or open synovectomy, but high rates of disease recurrence has driven the continued development of systemic therapies that retain intracellular target precision while minimizing adverse side effects. Systemic therapy alone, currently indicated for patients in whom surgical resection is not possible or considered highly morbid, has shown significant tumor size reduction and symptomatic relief for DTGCT; however, it fails to address mobility and functional decline due to disease progression and often is not considered a curative approach.

Growing evidence supports a combined approach of surgical resection with (neo)adjuvant systemic therapy. While further studies are necessary to investigate optimal treatment protocols of CSF1RI and surgery, early data suggest this strategy may improve outcomes and reduce tumor recurrence. As with other musculoskeletal tumors, patients with DTGCT are best treated in specialized centers under the guidance of multidisciplinary tumor boards. Further studies are needed to refine treatment algorithms and advance the standard of care for this challenging disease.
